# Functional Analysis of the Magnetosome Island in *Magnetospirillum gryphiswaldense*: The *mamAB* Operon Is Sufficient for Magnetite Biomineralization

**DOI:** 10.1371/journal.pone.0025561

**Published:** 2011-10-17

**Authors:** Anna Lohße, Susanne Ullrich, Emanuel Katzmann, Sarah Borg, Gerd Wanner, Michael Richter, Birgit Voigt, Thomas Schweder, Dirk Schüler

**Affiliations:** 1 Department Biologie I, Bereich Mikrobiologie, Ludwig-Maximilians-Universität München, LMU Biozentrum, Planegg-Martinsried, Germany; 2 Microbial Genomics and Bioinformatics Research Group, Max Planck Institute for Marine Microbiology, Bremen, Germany; 3 Department of Microbial Physiology, Institute of Microbiology, Ernst Moritz Arndt University, Greifswald, Germany; 4 Pharmaceutical Biotechnology Research Group, Institute of Pharmacy, Ernst Moritz Arndt University, Greifswald, Germany; Louisiana State University and A & M College, United States of America

## Abstract

Bacterial magnetosomes are membrane-enveloped, nanometer-sized crystals of magnetite, which serve for magnetotactic navigation. All genes implicated in the synthesis of these organelles are located in a conserved genomic magnetosome island (MAI). We performed a comprehensive bioinformatic, proteomic and genetic analysis of the MAI in *Magnetospirillum gryphiswaldense*. By the construction of large deletion mutants we demonstrate that the entire region is dispensable for growth, and the majority of MAI genes have no detectable function in magnetosome formation and could be eliminated without any effect. Only <25% of the region comprising four major operons could be associated with magnetite biomineralization, which correlated with high expression of these genes and their conservation among magnetotactic bacteria. Whereas only deletion of the *mamAB* operon resulted in the complete loss of magnetic particles, deletion of the conserved *mms6*, *mamGFDC*, and *mamXY* operons led to severe defects in morphology, size and organization of magnetite crystals. However, strains in which these operons were eliminated together retained the ability to synthesize small irregular crystallites, and weakly aligned in magnetic fields. This demonstrates that whereas the *mamGFDC*, *mms6* and *mamXY* operons have crucial and partially overlapping functions for the formation of functional magnetosomes, the *mamAB* operon is the only region of the MAI, which is necessary and sufficient for magnetite biomineralization. Our data further reduce the known minimal gene set required for magnetosome formation and will be useful for future genome engineering approaches.

## Introduction

The ability of magnetotactic bacteria (MTB) to orient in the earth's magnetic field is based on specific organelles, the magnetosomes. In the α-proteobacterium *Magnetospirillum gryphiswaldense* and related MTB, magnetosomes consist of magnetite (Fe_3_O_4_) crystals enclosed by a phospholipid membrane. This magnetosome membrane (MM) contains a specific set of >20 proteins, which direct the biomineralization of highly ordered crystals [1], [2], [3]. Synthesis of magnetosomes has recently emerged as a model for prokaryotic organelle formation and biomineralization [4], [5] In addition, magnetosomes represent biogenic magnetic nanoparticles with unique characteristics, which make them attractive for use in a wide range of biomedical and biotechnological applications [4], [6], [7]. Although the mechanism of magnetosome synthesis is not understood in detail, several recent studies revealed that the formation of functional magnetosomes depends on several steps, which include the invagination of MM vesicles from the inner membrane [8], [9], the transport of iron and crystallization of magnetite within these vesicles [10], and the assembly of mature crystals into a linear chain along a filamentous cytoskeletal structure [9], [11], [12], [13]. It has been also become clear that each of these steps is under strict genetic control. By proteomic analysis of *M. gryphiswaldense* (in the following referred to as MSR), genes encoding the MM-specific proteins were identified within a single genomic magnetosome island (MAI) [14], [15]. The functional significance of this region was confirmed by a comparative genomics approach, which revealed that magnetotaxis signature genes are predominantly located within the MAI [16]. Because of their general conservation in other cultivated and uncultivated α-proteobacterial MTB [3], [17], [18], [19] it has been suggested that the MAI was transferred horizontally [15], [16], [18], [20], [21]. This was further corroborated by the recent discovery of homologous gene clusters in the δ-proteobacteria *Desulfovibrio magneticus* RS-1 [22] and the multicellular magnetotactic prokaryote (MMP) [23], as well as in the deep-branching *Nitrospirae*-phylum [21]. In addition to all genes, so far implicated in magnetosome biomineralization, the MAI of MSR contains a number of genes with unknown functions and numerous transposase genes that account for >20% of the coding region [14]. Owing to frequent homologous recombinations between the numerous direct or inverted repeats associated with transposase genes, the MAI is genetically unstable, resulting in frequent spontaneous loss of the magnetic phenotype [15], [24]. In MSR all known magnetosome genes are comprised within four gene clusters known as *mms6*, *mamGFDC*, *mamAB*, and *mamXY* operons. First experimental indications for their functional significance in magnetosome formation came from the isolation of a non-magnetic mutant strain, which had lost 40 kb of the MAI by a spontaneous deletion that included the *mamAB*, *mms6* and *mamGFDC* operons [25]. Targeted deletion of the entire *mamGFDC* operon revealed that the small MamGFDC proteins, which account for >35% of all magnetosome-associated proteins, are not essential, but involved in size control, since mutant cells formed smaller and less regular magnetite crystals [26]. In a recent study by Murat *et al.* deletion analysis of the MAI in *M. magneticum* AMB-1 (referred to as AMB) revealed three regions, which are crucial for magnetite crystal formation [27]. Whereas the deletion of the R2 and R3 regions including parts of the *mamGFDC* and *mms6* operons led to severe defects in the size and morphology of the crystals, loss of the *mamAB* operon resulted in cells entirely devoid of magnetite crystals [27]. Only the deletion of *mamE*, *M*, *N*, *O*, *L*, *I*, and also of *mamQ* and *mamB*, if co-deleted with their respective duplicates outside the *mamAB* operon, entirely abolished magnetite synthesis. Non-magnetic cells were also observed upon deletion of this operon in MSR [25]. This suggested that only the *mamAB* operon may contains genes that are absolutely essential [27]. However, it has remained unknown whether this region is also sufficient for magnetosome biomineralization in the absence of other magnetosome genes, since possible genetic redundancy was suggested by the identification of genes, which are identical or similar to genes from *mamAB* operon and partially encoded within a “magnetosome islet” located elsewhere in the genome of AMB [28].

Despite morphological similarities between the strains AMB and MSR, previous studies suggested that function of orthologous genes might be somewhat distinct in these organisms depending on their different genetic context [8], since only about 50% of all genes are shared by the genomes of these two strains [16]. In particular, the MAI regions flanking the magnetosome operons show a divergent organization, gene content and were speculated to possibly harbor additional determinants for magnetosome formation [16], [18]. Here, we show that highly expressed and conserved genes within the MAI of MSR are mostly confined to the *mms6*, *mamGFDC*, *mamXY*, and *mamAB* operons. By deletion of these operons, either independently or in combination, we demonstrate that all four of them have crucial and partially overlapping functions in the synthesis of functional magnetosomes, whereas only the *mamAB* operon is absolutely essential for magnetite biomineralization. Intriguingly, even in the absence of all other three operons as well of further parts of the MAI, the *mamAB* operon proved sufficient to maintain synthesis of small magnetite crystals. A further motivation for this study was to explore the potential for reduction of dispensable or instable gene content from the residual MAI. By using an improved Cre-*lox*-based technique, we demonstrate that 115 kb of the MAI can be deleted without any consequences for growth, while 59 kb have no obvious function in magnetosome synthesis.

## Results

### Expression of MAI genes coincides with their conservation and operon localization

**Figure 1:**
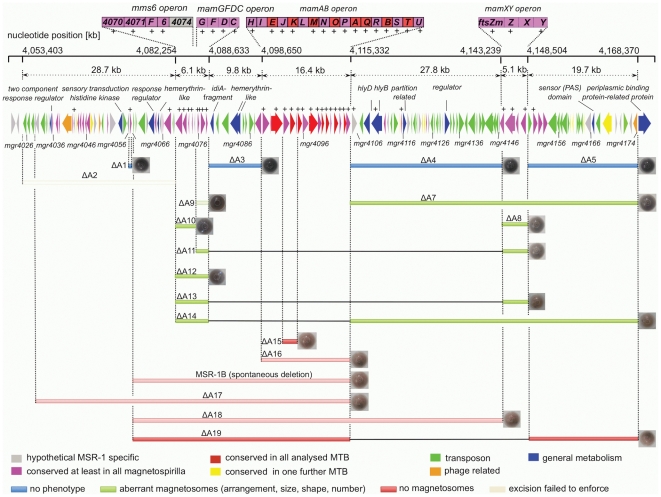
Molecular organization and characteristics of the MAI in *M. gryphiswaldense*. Extensions of deletions are shown by bars of different colors indicating the general phenotype. For overview, strains generated in previous studies are shown in semi-transparent color. The magnetite content of mutant strains is illustrated by the color of corresponding cell pellet. Degree of gene conservation is highlighted by different colors. Genes found expressed by proteomic analysis are indicated by “+”.

Besides numerous (>50) transposase and phage related genes, the *mam* and *mms* operons within the MAI are flanked by a number of ORFs, mostly annotated as hypothetical genes, which may represent either unrecognized determinants for magnetosome formation, genes with unknown different functions, or simply pseudogenes or misannotations. To tentatively distinguish between regions of predicted relevance and those not likely involved in magnetotaxis, we reasoned that putative magnetosome genes are expected (I) to lack strong prediction of other cellular functions, (II) to be highly conserved among MTB, and (III) to be expressed during magnetosome synthesis. We therefore reassessed functional annotation of the MAI against current databases. Only 12 of the MAI genes have functionally predicted homologs outside MTB (Fig. 1), which encode three hemerythrin-like proteins, putative regulatory proteins, secretion components, a sensory transduction histidine kinase, a partition-related protein, and an IdiA fragment (Table S1). To identify conserved genes, we tested by blastp analysis the presence of all genes from the MAI of MSR against all genomic information available from cultivated MTB (Fig. 1, Table S1). Genes that are highly conserved between several MTB were found mostly confined to the *mam* and *mms* operons, where ten ORFs (*mamE*, *K*, *M*, *O*, *A*, *Q*, *B*, T, and with lower similarity *mamI* and *mamP*) are conserved in all analyzed strains including MSR, AMB, *Desulfovibrio magneticus* RS-1, *M. magnetotacticum* MS-1, *Magnetococcus marinus* MC-1, and *Magnetovibrio blakemorei* MV-1. *MamE*, *I*, *K*, *M*, *O*, *P*, *A*, *Q*, *B* genes were also detected in the metagenomic MAI fragment Fos001, whereas a second metagenomic clone Fos002 lacks *mamI* but contains *mamT* [20]. *MamE*, *I*, *M*, *P*, *A*, *B*, and two *mamQ* homologs were also found in the incomplete MAI sequence of “*Candidatus* Magnetobacterium bavaricum” [21]. Nine ORFs have homologs in only one other MTB (Fig. 1), and 41 genes are shared by at least all magnetospirilla (Fig. 1). However, only 7 of these genes show positional conservation within the MAI of AMB, whereas the rest is located elsewhere in the genome in the latter strain. 22 genes, which are mostly confined to larger regions close to the putative boundaries of the MAI, are specific for MSR (i. e., have no homolog in any other organism), and appear less likely to represent determinants required for magnetosome formation. Thus, hypothetical genes outside the *mam* and *mms* operons are poorly conserved, with none of them found shared by all sequenced MTB.

To identify expressed products of ORFs encoded within the MAI, we performed proteomic analyses of magnetosomes, as well as intracellular soluble and membrane-enriched protein fractions of cells grown under magnetite forming conditions. In total, 923 proteins were identified by 1D LC–MS/MS analysis, or from spots detected on 2D gels. In summary, only 33 proteins encoded within the MAI were found expressed in the membrane or magnetosome fraction of MSR. These for instance include, with the exception of Mgr4074, MamI, MamL, and MamX, all proteins encoded by the *mamAB*, *mamGFDC*, *mms6*, and *mamXY* operons, whereas only seven genes outside the *mam* and *mms* operons were found expressed (*mgr4041*, *mamW*, *mgr4067*, *mgr4106*, *mgr4109*, *mgr4115*; *mgr4152*, Fig. 1; Table S1) as well as one gene barely inside the boundaries of the 130 kb region (*mgr4022*) [29]. With the exception of MamK, none of the MAI proteins was detected within the soluble protein fraction among the analyzed spots.

### Mutagenesis of MAI genes

**Figure 2:**
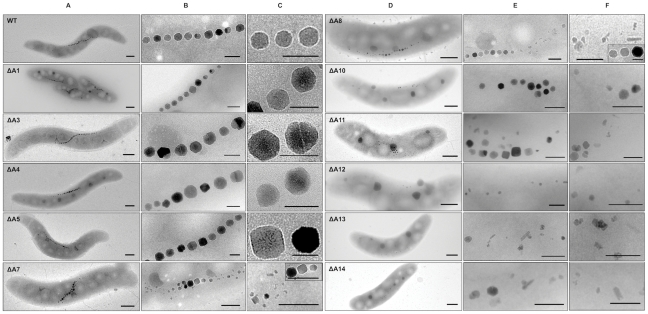
TEM micrographs of cells (A, D) and magnetosome morphologies (B, C, E, F) observed within the generated deletion mutants. Scale bar: 400 nm in A and D; 50 nm in B and C; 100 nm in E and F.

**Figure 3:**
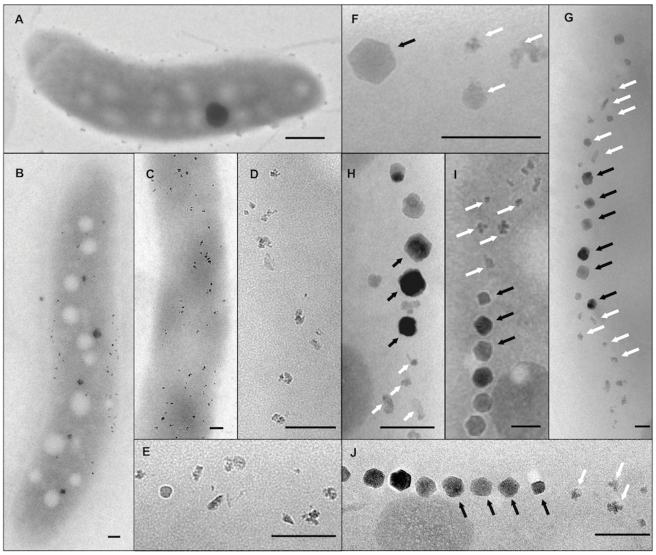
Representative TEM micrographs of magnetosome morphologies found in different cells of the ΔA8 deletion mutant. A) Cells without any electron-dense particles, B–E) irregularly shaped crystals lacking chain configuration, F–J) chains of regular crystals (black arrows), flanked by small particles with irregular morphologies (white arrows). Scale bar: 100 nm.

**Figure 4:**
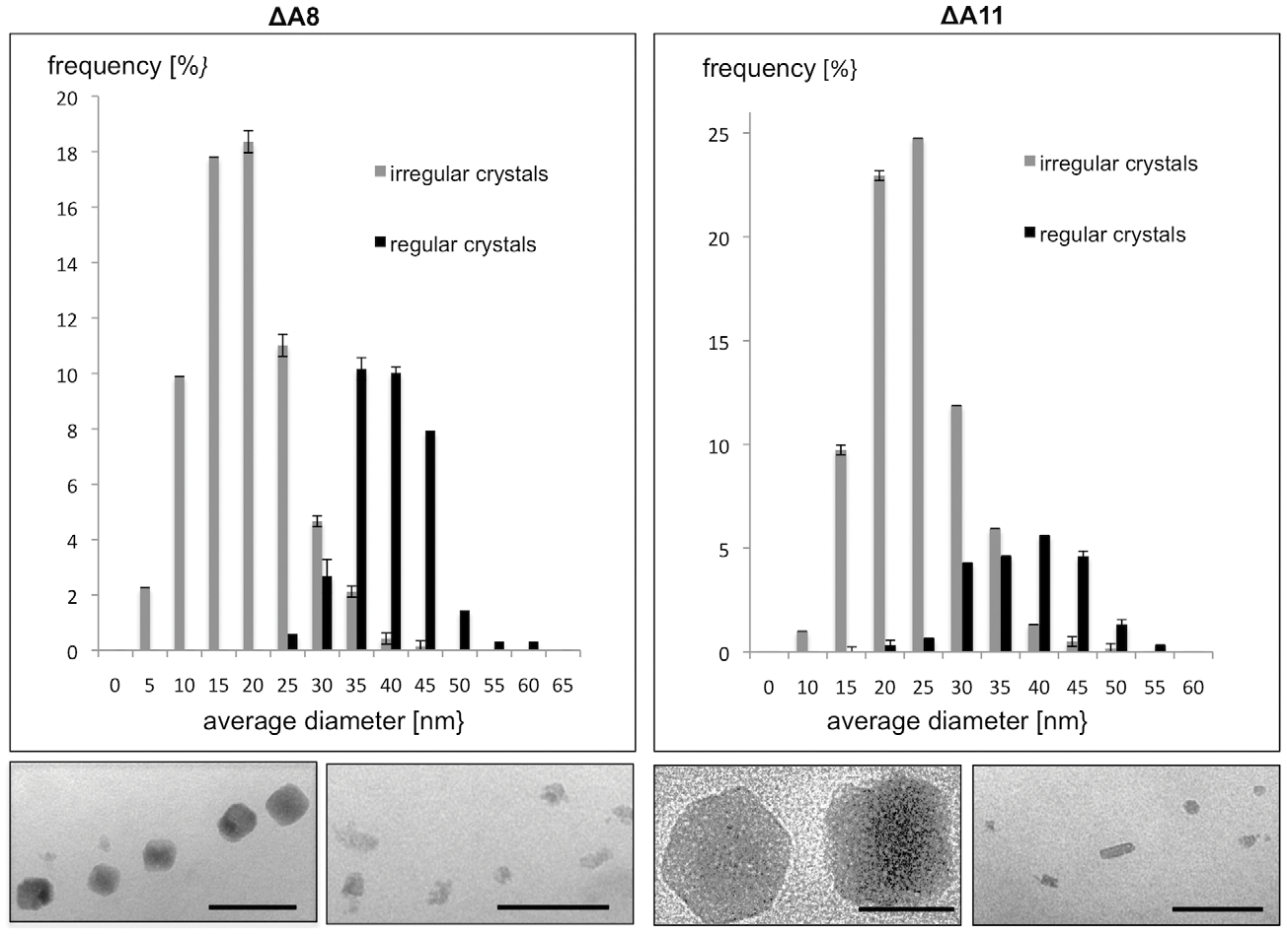
Magnetosome size distributions of electron dense particles within the mutants ΔA8 and ΔA11. Representative micrographs of corresponding crystal morphologies are shown. Scale bar: 100 nm.

By excluding putatively essential genes such as tRNA and rRNA genes, we predicted a core region of 115 kb from *mgr4026* to *mgr4074*, comprising 149 ORFs that are probably not important for central metabolic functions and including all so far known magnetosome genes. According to bioinformatic prediction and expression data, this region was divided into partially overlapping target regions for mutagenesis (Fig. 1). We constructed 13 mutant strains in which single or several of these targets were excised, resulting in deletions between 400 bp and 61 kb. Shorter deletions (up to 7 kb) were generated by allelic replacement (double crossover mediated by homologous recombination, Fig. S1A) [30], whereas Cre-*lox* excision (Fig. S1B; Fig. S2) [25], [31], was used for the construction of larger deletions between 5 and 53 kb. We noticed that success of deletion mutagenesis was not fully predictable. For instance, whereas we previously generated the ΔA17 deletion in the MSR-1B background [25], we failed to enforce deletion of parts of that region (ΔA2) in the WT background despite of repeated attempts. With few exceptions described below, all mutants including the longest deletion (ΔA14) extending over 58.9 kb exhibited WT-like growth, indicating that no central metabolic functions are encoded by deleted MAI genes. However, Cmag measurements and TEM of mutant strains revealed three different classes of phenotypes with respect to magnetosome formation: (I) Mutants that were unaffected in magnetosome formation, i. e. cells were virtually WT-like with respect to crystal appearance (shape, size, number per cell and alignment) including the long deletions ΔA3 (9.8 kb), ΔA4 (27.8 kb), and ΔA5 (19.7 kb), as well as *ΔmamW* (411 bp), eliminating a protein that was previously identified as associated with magnetosomes in MSR [15], [16]. (II) Mutants in which magnetosome formation was entirely abolished, as indicated by a pale pink to orange cell pellet (in contrast to the black appearance of the WT), lack of a magnetic response (Cmag = 0) and the absence of any electron dense particles. The non-magnetic mutants ΔA19, in which an additional 19.7 kb fragment was excised in the background of deletion mutant MSR-1B, and ΔA15 comprising the *mamJKL* genes, had in common a deletion of either the entire *mamAB* operon or parts of it, similar to strains MSR-1B, ΔA16, ΔA17 and ΔA18, which had been generated in previous studies [15], [25]. (III) A third class of mutant strains still exhibited a magnetic response, but cells were gradually affected in magnetosome biomineralization or assembly, resulting in fewer, smaller and irregular crystals or distorted chains (Fig. 2). Mutants of this class could be recognized by variable intensities of brownish color of colonies and cell pellets (Fig. 1). Single-operon deletions of *mms6* (ΔA10) and *mamXY* (ΔA8) showed a significantly reduced magnetic response, but still contained electron-dense particles with different sizes and shapes (Table 1). Strain ΔA10 had smaller crystals (Table 1) that were scattered throughout the cell or aligned in irregular, widely spaced “pseudo-chains” (i. e., with <10 crystals per chain; Fig. 2). Crystals between 25 and 30 nm were predominant, whereas particles larger than 50 nm were not observed, unlike WT crystals that were most frequently between 40 and 50 nm with a maximum size up to 70 nm (data not shown). Besides cubo-octahedral crystals also heterogeneous crystal shapes were observed (Fig. 2). Complementation with fragments comprising genes *mgr4072*, *mgr4073*, and *mgr4074* restored size, shape and alignment of crystals to WT range within about one third of the cells (data not shown). Strain ΔA8 had an inconsistent phenotype. TEM revealed a variety of magnetosome appearances between different cells, including those lacking any electron-dense particles (Fig. 3 A), and those with non-uniform, small crystals lacking any chain configuration (Fig. 3 B–F). Remarkably, many cells contained two distinct types of crystals: short chains of almost regular (i.e., cubicle-shaped) crystals, which were flanked by irregular particles with poorly defined morphologies (Fig. 3 G–K). Analysis of about 350 crystals from cells of the latter phenotype revealed that approximately 66% of the crystals were irregular and less electron dense, slightly elongate and poorly crystalline particles (Fig. 2). The different particles had distinct size distributions: Among irregular particles, sizes between 15 and 25 nm were most abundant, whereas the regular-shaped crystals had a maximum size of 60 nm, and diameters between 35 to 45 nm were most frequent among them (Fig. 4). The WT-like phenotype could be restored by transcomplementation with plasmid pmamXY containing the entire *mamXY* cluster (*mgr4147* to *mgr4150*; data not shown). A similar phenotype was observed for the mutant ΔA7 (Fig. 2) in which the deletion included the regions A4 and A5 in addition to the *mamXY* operon (Fig. 1; Table 1), resulting in an average crystal size of 23.5 nm. Crystal number per cell was not significantly affected in comparison to WT (Table 1). Operons whose single deletions had magnetosome phenotypes were also deleted in combination with each other. This was also achieved by modification of the previously described Cre-*lox* method [25] by using altered *lox* sequences [32] that enabled the construction of strains bearing multiple unmarked deletions by sequential rounds of insertions and excisions (Fig. S1). In strain ΔA12 the entire *mms6* operon was deleted in addition to the adjacent *mamGFDC* operon. This resulted in a stronger phenotype compared to its parent strain Δ*GFDC* [26], i. e. it formed even fewer and smaller magnetosomes that were aberrantly shaped and less regularly aligned (Fig. 2). The deletion of both operons also resulted in a particle size reduction of 52% compared to the WT, although crystals were only slightly smaller than in a deletion of *mms6* operon alone (Table 1). While crystal numbers per cell were only slightly reduced in comparison to the *mms6* operon mutant, the magnetic response of ΔA12 culture was markedly weaker (Cmag_[ΔA12]_ = 0.6; Table 1). The ΔA11 double deletion mutant of *mamXY* and *mamGFDC* operons showed a reduced Cmag (Cmag_[ΔA11]_ = 1.2; Table 1) and a phenotype as inconsistent as strain ΔA8 (Fig. 3). Compared to ΔA8, particles were smaller (Fig. 4), fewer per cell and less frequently aligned in chain-like structures (Fig. 2). Also, the number of crystals with regular morphology was reduced to 21.8%.

**Table 1:** Characteristics of MAI deletion mutants.

				Phenotypic characteristics		
Name of the strain	Deleted genes	Method of deletion	Extend of deletion	Cmag^a^	Average magnetosome size [nm]	Number of magnetosomes per cell
Wild type [53]	/	/	/	2.0±0.1	47.8−35.6^b^	34.3±8.4
ΔA1 (ΔmamW)	*mgr4057*	allelic replacement	411 bp	WT	WT (37.2±10.7)	WT (28.8±4.3)
ΔA2	*mgr4026* to *mgr4069*	Cre-*lox* two vectors	28,728 bp	/	/	/
ΔA3	*mgr4079* to *mgr4088*	Cre-*lox* two vectors	9,828 bp	WT	WT (41.2±13.7)	WT (27.8±4.7)
ΔA4	*mgr4106* to *mgr4146*	Cre-*lox* two vectors	27,795 bp	WT	WT (39.7±15.5)	WT (28.5±8.2)
ΔA5	*mgr4151* to *mgr4174*	Cre-*lox* two vectors	19,651 bp	WT	WT (35.0±14.2)	WT (29.9±8.6)
ΔA7	*mgr4106 to mgr4174*	Cre-*lox* two vectors	52,823 bp	Intermediate	Intermediate (23.5±15.9)	WT (35.0±8.2)
ΔA8 (ΔmamXY)	*mgr4147* to *mgr4150*	allelic replacement	5,077 bp	Intermediate	Intermediate (23.0±11.5)	WT (32.2±11.4)
ΔA9 (ΔGFDC) [26]	*mgr4075 to mgr4078*	allelic replacement	2,071 bp	Intermediate [26]	Intermediate [26]	WT [26]
ΔA10 (Δmms6 op)	*mgr4070* to *mgr4074*	allelic replacement	3,632 bp	Intermediate	Intermediate (19.7±6.9)	Intermediate (16.8±6.2)
ΔA11 (ΔmamGFDC_ ΔmamXY)	*mgr4075* to *mgr4078*; *mgr4147* to *mgr4150*	allelic replacement	7,148 bp	Intermediate	Intermediate (20.7±10.3)	Intermediate (25.3±6.0)
ΔA12 (Δmms6 op_ ΔmamGFDC)	*mgr4070* to *mgr4078*	allelic replacement	6,070 bp	Weak	Intermediate (18.4±6.0)	Intermediate (15.3±5.6)
ΔA13 (Δmms6 op_ ΔmamGFDC_ ΔmamXY)	*mgr4070* to *mgr4078*; *mgr4147* to *mgr4150*	allelic replacement	11,050 bp	Weak	Intermediate (19.3±8.1)	Weak (13.0±4.3)
ΔA14 (ΔA7_ Δmms6op_ ΔmamGFDC)	*mgr4106 to mgr4174; mgr4070 to mgr4078*	Cre-*lox* two vectors and allelic replacement	58,893 bp	Weak	Intermediate (19.7±7.7)	Weak (12.1±3.4)
ΔA15 (ΔmamJKL)	*mgr4092* to *mgr4094*	allelic replacement	2,656 bp	non magnetic	0	0
ΔA16 (mamAB#K7) [25]	*mgr4089* to *mgr4105*	Cre-*loxP* two vectors	16,362 bp	non magnetic	0	0
ΔA17 (MSR-1_SU12) [25]	*mgr4029* to *mgr4105*	Cre-*loxP* two vectors	61,000 bp	non magnetic	0	0
ΔA18 (MSR-1B mgr4058 to mgr4146) [25]	*mgr4058* to *mgr4146*	Cre-*loxP* two vectors	67,345 bp	non magnetic	0	0
ΔA19	*mgr4058 to mgr4105; mgr4151* to *mgr4175*	Cre-*loxP* two vectors	60,033 bp	non magnetic	0	0

a WT: no signiffcant difference to WT cells; Intermediate: 80-40% of WT characteristic; Weak: less than 40% of WT characteristic.

b Mean sizes were found slightly variable within a range between 48-35 nm due to minor variations of cultivation conditions and growth phase.

**Figure 5:**
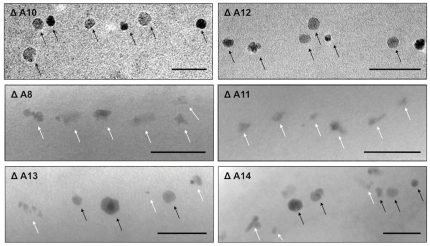
Comparison of magnetosome morphologies within several mutant strains of *M. gryphiswaldense*. Illustration of the combined effect on crystal morphology caused by stepwise excision of *mms6*, *mamGFDC* and *mamXY* operons. Micrographs show various distinct crystal morphologies within strains ΔA10 and ΔA12 (cubicle-shaped, black arrows) and ΔA8 and ΔA11 (elongate shaped, white arrows) that are coexistent within the mutants ΔA13 and ΔA14. Scale bar: 100 nm.

We also eliminated *mms6*, *mamGFDC*, and *mamXY* operons altogether using two approaches: While sequential triple deletion by allelic replacement of the three regions resulted in strain ΔA13, deletion of the *mamGFDC* and *mms6* operons in a parental background (ΔA7) that already lacked the entire right arm of the MAI (about 53 kb) containing the *mamXY* operon resulted in strain ΔA14 (Fig. 1). Remarkably, both strains still displayed a detectable, although weak magnetic response (Cmag_[ΔA13]_ = 0.3; Cmag_[ΔA14]_ = 0.5) and contained tiny misshapen electron dense crystallites (Fig. 2; Table 1). Crystal sizes were decreased to 54.8% of WT size and 84.8% of ΔA8 size, but were identical between ΔA13 and ΔA14 strains (Table 1). From all mutants, both strains ΔA13 and ΔA14 contained the fewest magnetosome number per cell (12–13 in average) and crystal shapes resembled the irregular morphologies found in strains ΔA7, ΔA8, ΔA10, ΔA11, and ΔA12. Thus, the phenotype of ΔA13 and ΔA14 is characterized by the coexistence of distinct particle morphologies found in the respective single operon deletion mutants (Fig. 5).

## Discussion

We performed a comprehensive investigation of the MAI in MSR by combined bioinformatic, proteomic and genetic analysis. With the exception of *mgr4041* and *mgr4106*, which are MSR-specific, all other genes from the 115 kb core region that were found expressed are also highly conserved in magnetospirilla or even all MTB. The majority of expressed genes (26 of 33) were localized within the *mms6*, *mamGFDC*, *mamAB*, and *mamXY* operons [25], [27]. These were also the only regions, which displayed a magnetosome phenotype upon their deletion. Thus, in contrast to previous observations in AMB [27], conservation and expression of MAI genes showed a strong correlation with a function in magnetosome formation.

We used a Cre-*lox* based method [25], [31], which allows the efficient excision of large fragments. The largest single deletion obtained by this method comprised 53 kb in strain ΔA7. Using modified *lox*-sites enabled multiple sequential rounds of marker-less deletions. This resulted in strains in which up to 59 kb were deleted, comprising about 50% of the MAI and encoding 78 ORFs. Despite of repeated attempts, no deletion of the A2 region (Fig. 1) was obtained. Whereas the ΔA17 (MSR_SU12) deletion was straightforwardly generated in the MSR-1B background in a previous approach [25], we failed to partially delete this region (ΔA2) in the WT background. It remains to be shown whether this was due to low efficiency, or if deletion of this region would be lethal only in the presence of the residual MAI genes.

The absence of detectable phenotypes apart from magnetosome formation in the deletion strains indicates that the MAI encodes no important functions for growth under laboratory conditions. Whereas less than 25% of the MAI region could be associated with magnetosome formation, more than 50% of the MAI seems to have no obvious functions. Remarkably, among the genes with no phenotype are several of the magnetospirilla-specific genes, such as *mgr4067*, *mgr4109*, *mgr4115*, *mgr4152*, and *mgr4057* (*mamW*), which had been previously implicated in magnetite synthesis because of its magnetosome expression [16]. We also failed to detect a phenotype for the two hemerythrin-like genes harbored within the deleted A3 region. Because of their MAI localization and the known functions of hemerythrins from other organisms in the sensing or transport of oxygen and iron, it was speculated that these proteins may play a role in magneto-aerotaxis and magnetosome formation [33], [34]. However, it cannot be excluded that their loss can be compensated by the numerous (i. e., 23) homologs encoded elsewhere in the genome. Taken together, although it remains possible that some deletion strains could show a phenotype under different growth conditions, or only in combination with other deletions, most of the genes flanking the identified magnetosome operons have no functional relevance and might just represent genetic “junk” or remnants from previous transfer events of the MAI.

Our deletion analysis confirmed several results of previous studies, in which the functional significance of several regions, such as *mamAB*, *mms6*, and *mamGFDC* were shown for AMB [27], and partially for MSR [25], [26]. However, despite of the high similarity of targeted genes, we also found several striking differences between the two organisms. One example is the conserved *mamXY* operon, which contains several magnetotaxis signature genes, and for which a key role was predicted mostly based on comparative genome analysis [16]. While MamY was recently implicated in MM biogenesis in AMB [35], *mamX* has similarity to the serine like proteases MamE and MamS, whereas MamZ is an ortholog of MamH and resembles permeases of the major facilitator superfamily. The FtsZ-like gene has homology to the tubulin-like protein, which is involved in cell division in many bacteria [36]. In contrast to the *mamXY* operon deletion in AMB, which did not show a strong effect [27], we found that *mamXY* genes have a crucial function in magnetite biomineralization of MSR. This is consistent with the results obtained by Ding et *al.*, who reported that the deletion of the *ftsZ-like* gene alone already resulted in the synthesis of smaller, predominantly superparamagnetic particles [37]. The deletion of all *mamXY* genes had an even stronger effect, which is different from all previously reported magnetosome phenotypes. Strikingly, all deletions including this operon had an inconsistent phenotype, which varied between different cells. In addition to size reduction, this was characterized by the coexistence of various distinct magnetosome morphologies within many single cells.

The deletion of genes from the *mms6* operon had slightly different effects in AMB and MSR too. Single deletion of the *mms6* gene in AMB already caused smaller and elongated crystals [38], thus resembling the R3 mutant constructed by Murat *et al.* [27], which comprised deletion of genes from both the *mms6* and *mamGFDC* operons. In contrast, 58% of crystals within cells of the single operon deletion mutant in MSR (strain ΔA10) still had cubicle-shaped appearance, whereas elongate crystals were absent from the mutants ΔA10 and ΔA12. Although the phenotypes cannot be directly compared, since the extents of deletions are not fully congruent, this might point towards slightly distinct functions of the homologous regions in AMB and MSR. In MSR co-deletion of the *mms6* operon together with *mamGFDC* in strain ΔA12 resulted in a further reduction of shape regularity and alignment of crystals, but only in a slight decrease of size, whereas the number of particles per cell was similar to strain ΔA10 (*Δmms6*). This argues for a certain functional overlap between the two operons, which is consistent with the high similarity between some of the encoded proteins, such as MmsF and MamF, which share 61% identity, and Mms6, which shares a conspicuous LG-rich motif with MamG and MamD [2], [39]. However, single operon mutant phenotypes suggest that genes of the *mms6* operon have a more pronounced effect on crystal size, number and alignment than *mamGFDC*, perhaps by direct binding to the surface of nascent crystallites through hydrophilic domains [40], or by enlarging the surface and curvature of MM vesicles, which spatially constrain the growth of magnetite crystals [26].

Intriguingly, even in the ΔA14 and ΔA13 strains, in which the *mms6*, *mamGFDC*, and *mamXY* operons were deleted in triple, magnetite formation was not entirely abolished and cells still weakly aligned in magnetic fields, although crystal sizes were further decreased and elongate crystals were present. Despite of a functional overlap in size control of magnetite crystals, the roles of the *mms6*, *mamGFDC*, and *mamXY* genes are not fully redundant, as indicated by the distinct morphologies found in their respective single operon deletions. While simultaneous excision of the *mamGFDC* and *mms6* operon lead to heterogeneous cubicle-shaped crystals, loss of *mamXY* operon lead to poorly crystallin and elongate crystals, which were also detected within the double deletion mutant of *mamXY* and *mamGFDC*. Interestingly, these effects are superimposed in the *mamGFDC*, *mms6*, *mamXY* triple deletion strains (ΔA13 and ΔA14), in which crystallites of both morphologies are present. Altogether, these observations indicate that the *mamGFDC*, *mms6* and *mamXY* operons have important and additive functions for the formation of regularly shaped crystals that are sufficiently large to be functional for interaction with the weak geomagnetic field [39], [41].

Consistent with observations for AMB [27], only the *mamAB* operon contains genes, which are essential for magnetosome formation. However, our data for the first time demonstrate that the *mamAB* operon is the only region of the MAI, which is necessary and sufficient to maintain magnetite biomineralization even in the absence of the *mamGFDC*, *mms6*, and *mamXY* clusters. Although it cannot be precluded that additional, so far unrecognized determinants might be encoded outside the MAI, this further reduces the minimal gene set, which is likely required for biomineralization. As the MamJ and MamK proteins were already shown to have roles in magnetosome chain assembly rather than in biomineralization [8], [42], the core set of MAI genes essential for magnetite biomineralization in MSR can be expected to be less than 15, and according to the identification of further non-essential genes in the *mamAB* operon of AMB (*mamA*, *H*, *U*, *V*, *P*, *T*, *R*, *S*) [27] this number is likely to shrink further.

Our results will be also useful for future genome reduction approaches. Comparable experiments in other bacteria have shown that large-scale deletions of target sequences are extremely powerful in engineering of strains optimized for biotechnological processes [43], [44], [45]. By stepwise removal of unnecessary or problematic genomic regions, in future approaches also strains of MSR can be engineered for the production of magnetosome particles, which may exhibit increased genetic stability due to the elimination of repeats and transposases, or might show improved growth or increased magnetosome yields because of reduced gene content. In summary, deletion analysis of MAI indicates that whereas only the *mamAB* operon is essential, different regions have important functions in control of size and morphology of magnetosomes. Thus, modular deletion or expression of various magnetosome genes and operons could be used for the production of engineered magnetic nanoparticles with tailored properties.

## Materials and Methods

### Bacterial strains, plasmids, and culture conditions

Bacterial strains and plasmids used in this study are listed in Table S2. *M. gryphiswaldense* strains were grown microaerobically in modified flask standard medium (FSM) at 30°C [46] and moderate agitation (120 rpm). *E. coli* strains were cultivated as previously described [47] and 1 mM DL-α, ε-diaminopimelic acid (DAP) was added to lysogeny broth media growing *E. coli* BW29427 (K. Datsenko and B. L. Wanner, unpublished data). Strains were routinely cultured on dishes with 1.5% (w/v) agar. For strains carrying recombinant plasmids, media were supplemented with 25 g/ml kanamycin (Km), 12 g/ml tetracycline (Tet), and 15 g/ml gentamicin (Gm) for *E. coli* strains, and 5 g/ml kanamycin, 5 g/ml tetracycline, and 20 g/ml gentamicin for *M. gryphiswaldense* strains, respectively. Blue-white screening was performed by adding 50 µg/ml X-Gluc (5-bromo-4-chloro-3-indoxyl-D-glucuronidase; AppliChem, Darmstadt, Germany) to FSM.

### Molecular and genetic techniques

The working draft of *M. gryphiswaldense* genome sequence (GenBank accession number No. CU459003) was used for primer design. Oligonucleotids (Table S3) were purchased from Sigma-Aldrich (Steinheim, Germany). Chromosomal DNA of *M. gryphiswaldense* was isolated as described previously [3]. Plasmids were constructed by standard recombinant techniques as described in detail in Materials and Methods S1. All constructs were sequenced on an ABI 3700 capillary sequencer (Applied Biosystems, Darmstadt, Germany), utilizing BigDye Terminator v3.1. Sequence data were analyzed with Software Vector NTI Advance® 11.5 (Invitrogen, Darmstadt, Germany).

### Analytical methods

Magnetic reaction of cells was checked by light microscopy applying a bar magnet.

Optical density and magnetic response (Cmag) of exponentially growing cells were measured photometrical at 565 nm as previously reported [48]. For Cmag messurement a magnetic field of approximately 70 millitesla was used [48]. As this field can possibly magnetize small magnetosomes in the superparamagnetic size range and cause artificially high Cmag readings, all putative magnetosome phenotypes were verified by transmission electron microscopy (TEM). For TEM analysis, exponential cells were 10-fold concentrated and adsorbed onto carbon-coated copper grids. Samples were viewed and recorded with a TECNAI FEI20 microscope (FEI, Eindhoven, Netherlands). Magnetosome crystals were analyzed with respect to size, shape and numbers per cell. Magnetosome crystals were scored for chain formation as described by [8]. For pictures of cell pellets, cells were cultivated anaerobic in FSM and 10^9^ cells were concentrated by centrifugation.

### Cell fractionation, protein digestion, mass spectrometry, and data analysis

For proteomic analysis *M. gryphiswaldense* WT was grown in microaerobic 1-liter batch cultures and cell fractions (membrane-enriched, soluble, and magnetosomes) were prepared as previously described [2], [29]. Soluble proteins were separated in 2D PAGE (pH 4–7 and 3–10). Analysis of 2D gels including relative quantification was done with the Delta2D software (Decodon, Greifswald, Germany). Protein spots were cut from 2D gels, transferred into microtiter plates, and tryptically digested using the Ettan Spot Handling Workstation (GE Healthcare, Munich, Germany). Mass spectra of protein fragments were measured by MALDI-TOF-MS/MS using a Proteome Analyzer 4800 (Applied Biosystems, Munich, Germany). The parameters for measurements were set as described in [49]. The spectra were searched against the published genome sequence from *M. gryphiswaldense* by using the JCoast 1.6 software [50], and proteins were identified using the Mascot search engine. For analysis of magnetosomes and membrane proteins, gel lanes obtained from 1D-SDS-PAGE were cut into 10 equal slices. Gel slices were digested manually with trypsin and analysed by LC coupled mass spectrometry performed as described by [51]. Relative quantification of membrane proteins was based on spectral counting using Scaffold [52].

## Supporting Information

Figure S1 **Schematic illustration of methods for generation of deletions within the MAI.** (A) Allelic replacement of target genes using double cross-over followed by removal of selection marker with Cre-*lox* mediated excision. (B) Cre-*lox* recombination using the modified sequences *lox71* and *lox66* for specific excision of large chromosomal regions and construction of marker-less mutant strains. After excision the modified lox* sequence remains in the genome, but is poorly recognized by Cre recombinase making multiple recombination events possible.
(TIF)

Figure S2 **Constructed suicide plasmids (pAL01 to pAL11_term) for integration of modified** ***lox*** **sequences.** Regions (AL01 to AL11) within the MAI of *M. gryphiswaldense* used for site-specific plasmid insertion via homologous recombination to enable subsequent excision between *lox* sites of double insertions.
(TIF)

Table S1 Strains and plasmids used in this study.
(DOC)

Table S2 DNA oligonucleotides used in this work.
(DOC)

Table S3 Annotation and characteristics of MAI genes of *M. gryphiswaldense*.
(DOC)

Materials and Methods S1 Construction of integrative plasmids and deletion mutagenesis/Conjugation experiments.
(DOC)
