# Supplementary figures and images for: Functional Analysis of the Magnetosome Island in Magnetospirillum gryphiswaldense: The mamAB Operon Is Sufficient for Magnetite Biomineralization
Source: PLoS One. 2011 Oct 17;6(10):e25561. doi: 10.1371/journal.pone.0025561 (PMC3197154; doi:10.1371/journal.pone.0025561)

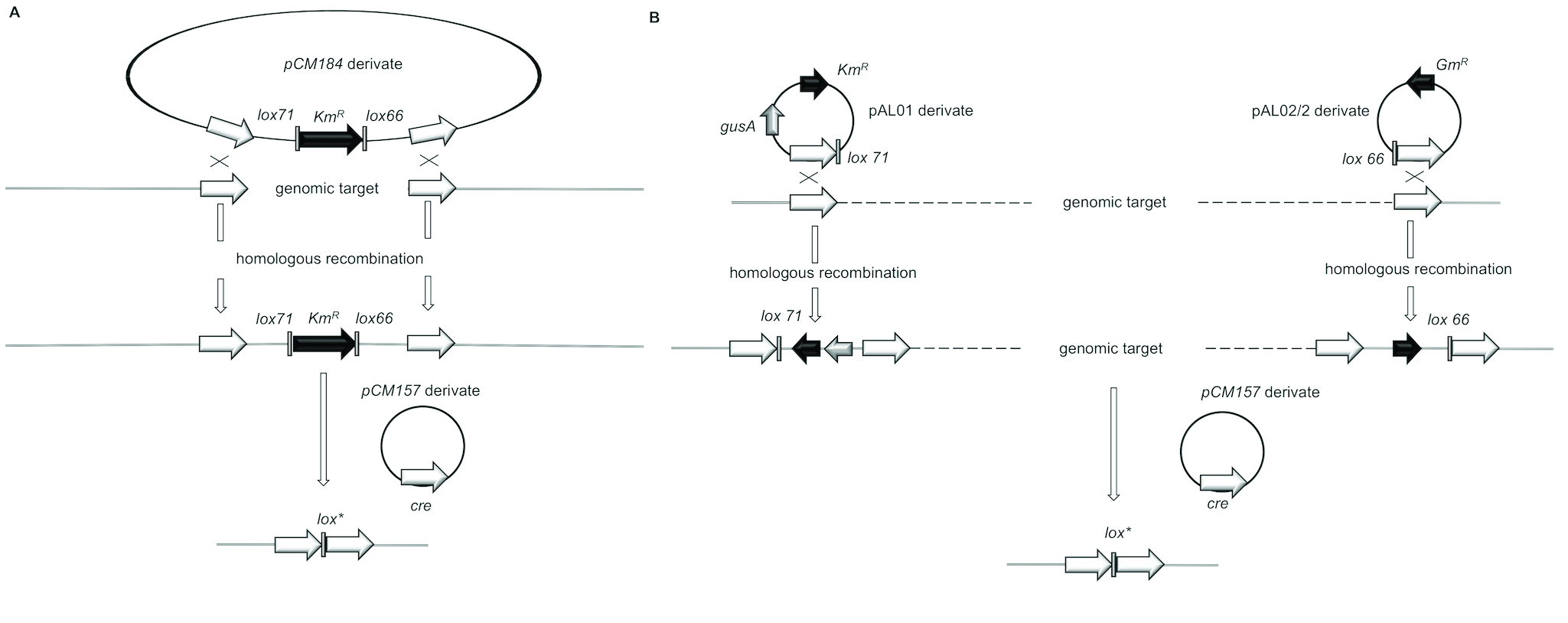

Supplement: Figure S1 — Schematic illustration of methods for generation of deletions within the MAI. (A) Allelic replacement of target genes using double cross-over followed by removal of selection marker with Cre-lox mediated excision. (B) Cre-lox recombination using the modified sequences lox71 and lox66 for specific excision of large chromosomal regions and construction of marker-less mutant strains. After excision the modified lox* sequence remains in the genome, but is poorly recognized by Cre recombinase making multiple recombination events possible. (TIF) [file pone.0025561.s001.tif]

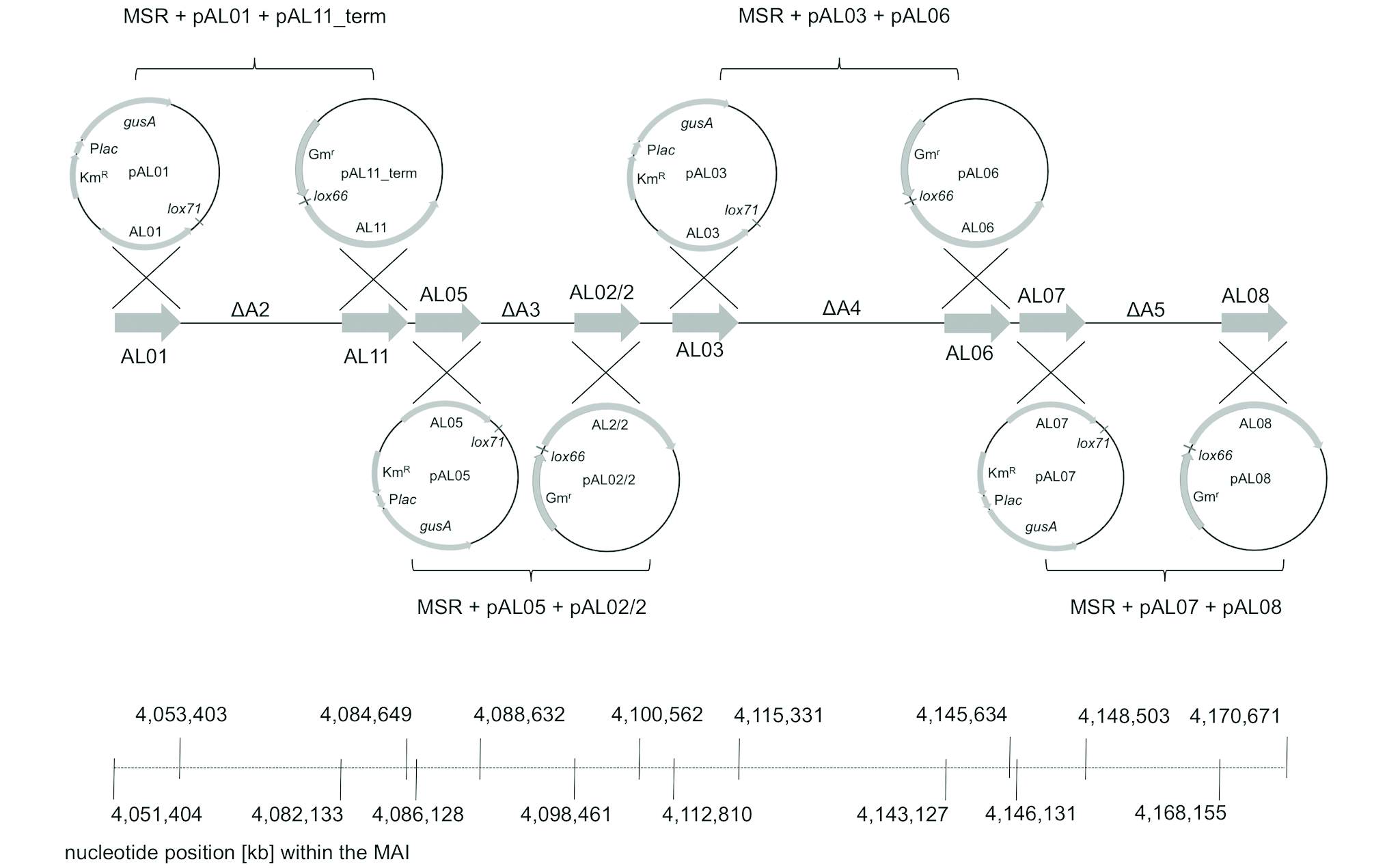

Supplement: Figure S2 — Constructed suicide plasmids (pAL01 to pAL11_term) for integration of modified lox sequences. Regions (AL01 to AL11) within the MAI of M. gryphiswaldense used for site-specific plasmid insertion via homologous recombination to enable subsequent excision between lox sites of double insertions. (TIF) [file pone.0025561.s002.tif]
