# Supplementary material for: Functional Analysis of the Magnetosome Island in Magnetospirillum gryphiswaldense: The mamAB Operon Is Sufficient for Magnetite Biomineralization
Source: PLoS One. 2011 Oct 17;6(10):e25561. doi: 10.1371/journal.pone.0025561 (PMC3197154; doi:10.1371/journal.pone.0025561)
Supplement: Table S1 — Strains and plasmids used in this study. (DOC) [file pone.0025561.s003.doc]

**Table S1.** Annotation and characteristics of MAI genes of *M. gryphiswaldense.*

| Genomic Location | Length [bp] | Annotation | Blastp hits for AMB/MS/MC/RS/MV* | Hits outside MTB (E-value / Organism)* | Protein expression/ Reference |
| --- | --- | --- | --- | --- | --- |
| *mgr4024* | 496 | hypothetical protein | - / - / - / - / nf | / | - |
| *mgr4025* | 266 | hypothetical protein | - / - / - / - / nf | / | - |
| *mgr4029* | 170 | hypothetical protein | - / - / - / - / nf | / | - |
| *mgr4030* | 283 | hypothetical protein | - / 7e-09 / 1e-153 / 5e-39 | 7e-09 / *Thermomicrobium roseum* DSM 5159 | - |
| *mgr4033* | 77 | hypothetical protein | - / - / - / - / nf | / | - |
| *mgr4034* | 71 | hypothetical protein | - / - / - / - / nf | / | - |
| *mgr4035* | 163 | two-component response regulator | 4e-23 / 1e-19 / 3e-09 / 7e-09 / nf | 1e-15 / *Caulobacter crescentus* NA1000 | - |
| *mgr4036* | 106 | hypothetical protein | 1e-08 / 2e-15 / - / - / nf | 2e-20 / *Pseudomonas putida* BIRD-1 | - |
| *mgr4037* | 199 | hypothetical protein | 3e-77 / 4e-75 / - / - / 5e-92 | 2e-9 / *Polaromonas sp*. JS666 | - |
| *mgr4038* | 145 | structural protein | 7e-77 / 8e-76 / - / - / nf | 5e-34 / *Desulfovibrio vulgaris str. Hildenborough* | - |
| *mgr4039* | 766 | phage-related | 0.0 / 0.0 / - / 1e-34 / 0.0 | 2e-164 / *Methylobacillus flagellatus* KT | - |
| *mgr4040* | 144 | hypothetical protein | 6e-74 / 3e-74 / -/ - / 2e-41 | 4e-38 / *Burkholderia vietnamiensis* G4 | - |
| *mgr4041* | 82 | hypothetical protein | - / - / - / - / nf | 2e-18 / *Candidatus* Accumulibacter phosphatis clade IIA str. UW-1 | + ^d^ |
| *mgr4042* | 137 | plasmid stability like | - / 3e-6 / - / - / nf | 8e-41/ *Polaromonas sp.* JS666 | - |
| *mgr4043* | 200 | hypothetical protein | 5e-97 / 4e-96 / - / - / 4e-47 | 8e-58 / *Burkholderia vietnamiensis* G4 | - |
| *mgr4044* | 72 | hypothetical protein | 1e-14 / 3e-15 / - /- / nf | / | - |
| *mgr4045* | 136 | hypothetical protein | 6e-73 / 2e-71 / - / - / nf | / | - |
| *mgr4046* | 270 | hypothetical protein | 9e-27 / 3e-25 / 3e-15 / - / nf | 3e-31 / α-proteobacterium BAL199 | - |
| *mgr4047* | 102 | hypothetical protein | 4e-13/ 9e-19 / - / - / nf | 1e-08 / *Rhodospirillum centenum* SW | - |
| *mgr4048* | 67 | hypothetical protein | - / 4e-17 / - / - / nf | 2e-21 / α-proteobacterium BAL199 | - |
| *mgr4049* | 138 | hypothetical protein | - / - / - / - / nf | / | - |
| *mgr4050* | 186 | phage-related | - / 1e-35 / 5e-14 / - / nf | 9e-64 / α-proteobacterium BAL199 | - |
| *mgr4052* | 116 | hypothetical protein | 3e-51 / 2e-51 / - / - / nf | / | - |
| *mgr4053* | 410 | hypothetical protein | - / - / - / - / nf | 3e-140 / *Mesorhizobium loti* MAFF303099 | - |
| *mgr4054* | 340 | sensory transduction histdine kinase | 1e-75 / 3e-73 /- / - / nf | 7e-61 / δ-proteobacterium MLMS-1 | - |
| *mgr4056* | 378 | hypothetical protein | - / - / - / - / nf | / | - |
| *mgr4057* | 138 | MamW | 1e-44 / 7e-45 / - / - / nf | / | + ^b, c, d^ |
| *mgr4061* | 342 | hypothetical protein | - / - / - / - / nf | / | - |
| *mgr4062* | 421 | two-component response regulator | e-122 / 2e-63 / - / - / nf | 1e-13 / *Asticcacaulis excentricus* CB 48 | - |
| *mgr4063* | 161 | hypothetical protein | 2e-30 / 4e-31 / - / - / nf | / | - |
| *mgr4064* | 159 | hemerythrin-like | 5e-58 / 8e-59/ 1e-10 / 1e-8 / nf | 3e-12 / *Candidatus* Methanoregula boonei 6A8 | - |
| *mgr4065* | 55 | hypothetical protein | - / - / - / - / nf | / | - |
| *mgr4066* | 109 | hypothetical protein | 7e-13 / 5e-13 / - / - / nf | / | - |
| *mgr4067* | 503 | pentapeptide repeat containing protein | 1e-172 / 1e-173 / - /- / nf | 5e-27 / *Anaerotruncus colihominis* DSM 17241S101 | + |
| *mgr4069* | 85 | hypothetical protein | - / - / - / - / nf | / | - |
| *mgr4070* | 449 | TPR-like protein | 1e-136 / 1e-135 / - / - / nf | 2e-22 / *Rhodospirillum rubrum* ATCC 11170 | + |
| *mgr4071* | 347 | hypothetical protein | 1e-86 / 2e-10 / - / - / nf | 4e-5 / *Starkeya novella* DSM 506 | + |
| *mgr4072* | 124 | MmsF | 7e-44 / 1e-44 / 4e-21 / - /8e-27 | 1e-5 / *Clostridium scindens* ATCC 35704 | + ^b, d^ |
| *mgr4073* | 136 | Mms6 | 2e-16/ 2e-16/ - / - / >1e-5 | / | + ^b, d^ |
| *mgr4074* | 90 | hypothetical protein | - / - / - / - / nf | / | - |
| *mgr4075* | 111 | MamG | 6e-9 / 1e-7 / - / - / nf | / | + ^b^ |
| *mgr4076* | 111 | MamF | 1e-42 / 1e-42 / 8e-20 / - / 9e-25 | 1e-5 / *Blautia hydrogenotrophica* DSM 10507 | + ^b, d^ |
| *mgr4077* | 314 | MamD | 2e-89 / 5e-90 / 7e-14 / - / 7e-5 | / | + ^a, b, d^ |
| *mgr4078* | 125 | MamC | 5e-21 / 5e-21 / 3e-07 / - / 1e-6 | / | + ^a, b, d^ |
| *mgr4079* | 278 | IdiA-fragment | 1e-82 / 1e-82 / - / - /nf | 1e-87 / *Synechococcus sp*. JA-2-3B'a(2-13) | - |
| *mgr4082* | 524 | hemerythrin-like | 6e-83/ e-105/ 7e-09 / 3e-10/ nf | 8e-13 / *Colwellia psychrerythraea* 34H | - |
| *mgr4083* | 150 | hemerythrin-like | 2e-83/ 1e-83 / 1e-11 / 2e-10/ nf | 3e-14 / *Candidatus Koribacter versatilis Ellin345* | - |
| *mgr4088* | 415 | hypothetical protein | 0.0 / 0.0 / - / - / nf | 2e-64 / *Bradyrhizobium sp.* BTAi1 | - |
| *mgr4089* | 428 | MamH | 0.0 / 3e-87 / 1e-116 / - /1e-141 | 1e-36 / *Chlorobium luteolum* DSM 273 | + |
| *mgr4090* | 77 | MamI | 3e-15 / 3e-15 /3e-11 / - / 2e-09 | / | - |
| *mgr4091* | 772 | MamE | 0.0 / 0.0 / 7e-57 / 1e-34 / 3e-119 | 1e-37 / *Rhodopirellula baltica* SH 1 | + ^a, b, d^ |
| *mgr4092* | 426 | MamJ | 2e-74 / 2e-74 / - / - / nf | / | + ^b, d^ |
| *mgr4093* | 360 | MamK | 0.0 / 0.0 / 3e-99 / 1e-65 / 1e-101 | 3e-92 / *Desulfurivibrio alkaliphilus* AHT2 | + ^d^ |
| *mgr4094* | 123 | MamL | 8e-32 / 1e-19 /- / - / nf | / | - |
| *mgr4095* | 318 | MamM | 1e-173 / 1e-173 / 1e-75 / 3e-35 / 1e-95 | 7e-33 / *Thermoanaerobacter sp*. X514 | + ^b, d^ |
| *mgr4096* | 437 | MamN | 0.0 / 0.0 / - / - / 4e-99 | 4e-46 / *Clostridium botulinum* H04402 065 | + ^b^ |
| *mgr4097* | 632 | MamO | 0.0 / 0.0 / 1e-78 / 3e-13 / e-148 | 4e-13 / *Acidimicrobium ferrooxidans* DSM 10331 | + ^b, d^ |
| *mgr4098* | 270 | MamP | 1e-108 / 1e-108 / 8e-34 / - / 2e-57 | 3e-4 / *Legionella pneumophila str. Corby* | + |
| *mgr4099* | 217 | MamA | 1e-113 / 1e-113 / 3e-37 / 1e-09 / 2e-49 | 2e-15 / *Microscilla marina* ATCC 23134 | + ^a, b, d^ |
| *mgr4100* | 272 | MamQ | 1e-111 / 1e-110 / 1e-37 / 2e-53 / 2e-48 | 1e-17 / *Bacillus sp*. NRRL B-14911 | + ^b, d^ |
| *mgr4101* | 72 | MamR | 1e-30 / 6e-31 / - / - / 3e-07 | / | + ^b, d^ |
| *mgr4102* | 297 | MamB | e-159 / e-159 / 2e-79 / 2e-36 / 2e-92 | 8e-44 / Natranaerobius thermophilus JW/NM-WN-LF | + ^a, b, d^ |
| *mgr4103* | 180 | MamS | 4e-60 / 2e-60 / 5e-13 / - / 5e-27 | / | + ^b, d^ |
| *mgr4104* | 174 | MamT | 2e-86/ 2e-82 /4e-26 / 9e-05 / 1e-40 | / | + ^b, d^ |
| *mgr4105* | 297 | MamU | 1e-114 / 1e-116 / - / - / nf | 2e-36 / *Azospirillum sp.* B510 | + |
| *mgr4106* | 411 | hypothetical protein | - / - / - / - / nf | / | + |
| *mgr4108* | 458 | HlyD secretion protein | 4e-38 / 1e-44 / 5e-67 / 5e-32 / 7e-60 | 1e-77 / *Bradyrhizobium japonicum* USDA 110 | - |
| *mgr4109* | 738 | HlyB secretion protein | 1e-72 / e-133 / 0.0 / e-128 | 0.0 / *Pseudomonas aeruginosa* | + ^d^ |
| *mgr4110* | 168 | Gp28 | 8e-04/ 2e-04 / - / - / nf | 4e-14 / *Polaromonas sp.* JS666 | - |
| *mgr4111* | 161 | hypothetical protein | - / - / - / - / nf | / | - |
| *mgr4114* | 69 | hypothetical protein | - / 7e-10 / - / - / nf | / | - |
| *mgr4115* | 115 | hypothetical protein | 2e-09/ 2e-08 / - / - / nf | / | + ^d^ |
| *mgr4116* | 250 | partition-related protein | 2e-13 / 1e-15 / 2e-06 / 5e-06 / nf | 1e-82 / *Azospirillum sp*. B510 | - |
| *mgr4117* | 216 | hypothetical protein | - / - / - / - / nf | 9e-50 / *Azospirillum sp*. B510 | - |
| *mgr4121* | 58 | hypothetical protein | - / - / - / - / nf | / | - |
| *mgr4122* | 140 | hypothetical protein | - / - / 2e-43 / - / nf | 1e-37 / *Aromatoleum aromaticum* EbN1 | - |
| *mgr4123* | 74 | hypothetical protein | - / - / 4e-25 / - / nf | 8e-22 / *Chlorobium phaeobacteroides* DSM 266 | - |
| *mgr4124* | 130 | hypothetical protein | 2e-50 / 3e-51 / 1e-40 / - / nf | 3e-28 / *Pseudomonas fluorescens* WH6 | - |
| *mgr4125* | 155 | hypothetical protein | - / - / - / - / nf | / | - |
| *mgr4131* | 77 | hypothetical protein | - / - / - / - / nf | 1e-20 / *Rhodoferax ferrireducens* T118 | - |
| *mgr4132* | 398 | regulator protein | 1e-130 / e-132 / - / - / nf | 2e-07 / *Hirschia baltica* ATCC 49814 | - |
| *mgr4140* | 93 | hypothetical protein | - / - / - / - / nf | / | - |
| *mgr4146* | 98 | hypothetical protein | - / - / - / - / nf | / | - |
| *mgr4147* | 323 | FtsZm | e-124 / e-124 / 6e-82 / 2e-79 / nf | 1e-119 / *Candidatus* Puniceispirillum marinum IMCC1322 | + ^d^ |
| *mgr4148* | 661 | MamZ | 0.0 / 0.0 / 1e-163 / - / 1e-128 | 4e-35 / *Variovorax paradoxus* EPS | + |
| *mgr4149* | 269 | MamX | 1e-117 / 1e-117 / 3e-34 / - / 3e-14 | / | - |
| *mgr4150* | 371 | MamY | 1e-139 / e-140 / - / - / 2e-17 | / | + ^d^ |
| *mgr4152* | 326 | hypothetical protein | 1e-154 / 1e-156 / - / - / 2e-111 | 7e-51 / *Chthoniobacter flavus* Ellin428 | + |
| *mgr4153* | 308 | hypothetical protein | 1e-141 / 1e-143 / - / - / 7e-63 | 1e-19 / *Spirosoma linguale* DSM 74 | - |
| *mgr4154* | 299 | hypothetical protein | 1e-140 / 1e-139 / 5e-134 / - / 9e-76 | / | - |
| *mgr4160* | 115 | hypothetical protein | 3e-17 / - / - / - / nf | / | - |
| *mgr4161* | 87 | hypothetical protein | - / 6e-47 / - / - / nf | / | - |
| *mgr4165* | 58 | hypothetical protein | 3e-10 / - / - / - / nf | / | - |
| *mgr4166* | 422 | hypothetical protein | 2e-74 / 2e-63 / - / - / nf | 5e-14 / α-proteobacterium BAL199 | - |
| *mgr4167* | 165 | sensor (PAS) domain | 4e-58 / 3e-43 / 4e-12 / 7e-07 / nf | 2e-45 / *Roseibium sp*. TrichSKD4 | - |
| *mgr4169* | 699 | hypothetical protein | 1e-112 / - / - / - /nf | 3e-58 / *Cellvibrio japonicus* Ueda107 | - |
| *mgr4170* | 133 | hypothetical protein | - / - / - / - / nf | / | - |
| *mgr4171* | 112 | hypothetical protein | - / - / - / - / nf | 2e-15 / *Hoeflea phototrophica* DFL-43 | - |
| *mgr4173* | 273 | hypothetical protein | - / - / - / - / nf | / | - |
| *mgr4174* | 190 | phage-related protein | 1e-21 / 5e-30 / 6e-07 / 6e-17 / nf | 8e-37 / *Paracoccus denitrificans* PD1222 | - |

*Homologs in magnetotactic or non magnetotactic bacteria were identified by NCBI database search engine with an e-value

threshold of <1e-05.

^a^ [10], ^b^ [11], ^c^ [12], ^d^ [13]

10. Grünberg K, Wawer C, Tebo BM, Schüler D (2001) A large gene cluster encoding several magnetosome proteins is conserved in different species of magnetotactic bacteria. Appl Environ Microbiol 67: 4573-4582.

11. Grünberg K, Müller EC, Otto A, Reszka R, Linder D, et al. (2004) Biochemical and proteomic analysis of the magnetosome membrane in Magnetospirillum gryphiswaldense. Appl Environ Microbiol 70: 1040-1050.

12. Ullrich S, Kube M, Schübbe S, Reinhardt R, Schüler D (2005) A hypervariable 130-kilobase genomic region of Magnetospirillum gryphiswaldense comprises a magnetosome island which undergoes frequent rearrangements during stationary growth. J Bacteriol 187: 7176-7184.

13. Uebe R, Voigt B, Schweder T, Albrecht D, Katzmann E, et al. (2010) Deletion of a fur-like gene affects iron homeostasis and magnetosome formation in Magnetospirillum gryphiswaldense. J Bacteriol 192: 4192-4204.
