# Supplementary material for: Functional Analysis of the Magnetosome Island in Magnetospirillum gryphiswaldense: The mamAB Operon Is Sufficient for Magnetite Biomineralization
Source: PLoS One. 2011 Oct 17;6(10):e25561. doi: 10.1371/journal.pone.0025561 (PMC3197154; doi:10.1371/journal.pone.0025561)
Supplement: Table S2 — DNA oligonucleotides used in this work. (DOC) [file pone.0025561.s004.doc]

**Table S2.** Strains and plasmids used in this study.

| Strains and plasmids | Description | References |
| --- | --- | --- |
| *M. gryphiswaldense* strains |  |  |
| MSR-1 R3/S1 | Rif^r^ Sm^r^, spontaneous mutant | [3] |
| MSR-1B | Spontaneous mutant, lacking 40,385 kb genomic region | [6] |
| ΔmamAB#K7 | Δ*mamAB* | [5] |
| MSR-1B mgr4058tomgr4146 | MSR-1B range of excision from *mgr4058* to *mgr4146* | [5] |
| MSR_SU12 | Δ*mamAB* with deletion to *mgr4029* | [5] |
| ΔGFDC | Δ*mamGFDC* | [2] |
| ΔmamJKL | Δ*mamJKL* | this study |
| MSR+pAL01 | MSR-1 R3/S1 (pAL01), Km^r^ | this study |
| MSR+pAL01+pAL02/2 | MSR-1 R3/S1 (pAL01, pAL02/2), Km^r^, Gm^r^ | this study |
| MSR+pAL01+pAL11_term | MSR-1 R3/S1 (pAL01, pAL11_term), Km^r^, Gm^r^ | this study |
| MSR+pAL03 | MSR-1 R3/S1 (pAL03), Km^r^ | this study |
| MSR+pAL03+pAL06 | MSR-1 R3/S1 (pAL03, pAL06), Km^r^, Gm^r^ | this study |
| MSR+pAL03+pAL08 | MSR-1 R3/S1 (pAL03, pAL08), Km^r^, Gm^r^ | this study |
| MSR+pAL05 | MSR-1 R3/S1 (pAL05), Km^r^ | this study |
| MSR+pAL05+pAL02/2 | MSR-1 R3/S1 (pAL02/2, pAL05), Km^r^, Gm^r^ | this study |
| MSR+pAL07 | MSR-1 R3/S1 (pAL07), Km^r^ | this study |
| MSR+pAL07+pAL08 | MSR-1 R3/S1 (pAL07, pAL08), Km^r^, Gm^r^ | this study |
| ΔA2 | MSR-1 R3/S1 range of excision from *mgr4026* to *mgr4069* | this study |
| ΔA3 | MSR-1 R3/S1 range of excision from *mgr4079* to *mgr4088* | this study |
| ΔA4 | MSR-1 R3/S1 range of excision from *mgr4106* to *mgr4146* | this study |
| ΔA5 | MSR-1 R3/S1 range of excision from *mgr4151* to *mgr4174* | this study |
| ΔA7 | MSR-1 R3/S1 range of excision from *mgr4106* to *mgr4174* | this study |
| ΔA8 | Δ*mamXY* | this study |
| ΔA10 | Δ*mms6* operon | this study |
| ΔA11 | Δ*mamXY,* Δ*mamGFDC* | this study |
| ΔA12 | Δ*mms6* operon*,* Δ*mamGFDC* | this study |
| ΔA13 | Δ*mms6* operon*,* Δ*mamGFDC,* Δ*mamXY* | this study |
| ΔA14 | Δ*A7* with deletion of *mms6* and *mamGFDC* operon | this study |
| ΔA19 | MSR-1B with deletion from *mgr4151* to *mgr4175* | this study |
| ΔA10_pCDS52_mms6_mmsF | Δmms6 operon (p*CDS52_mms6_mmsF*), Km^r^ | this study |
| ΔA10_pBBR-MCS2 | Δ*mms6* operon (pBBR-MCS2), Km^r^ | this study |
| ΔA8_pmamXY | Δ*mamXY* (pmamXY), Km^r^ | this study |
| MSR-1_pmamXY | MSR-1 R3/S1 (pmamXY), Km^r^ | this study |
| *E. coli* strain |  |  |
| *E. coli* BW29427 | *thrB1004 pro thi rpsL hsdS lacZDM15 RP4-1360D(araBAD)567DdapA* | Datsenko and Wanner (unpublished) |
| *E. coli* DH5a | *1341::[ermpir(wildtype)]trahsdR17 recA1-endA1gyrA96thi-1relA1* | Invitrogen |
| Plasmids |  |  |
| pJet1.2 | Ap^r^, *eco47IR*, *rep* (pMB-1) | Fermentas |
| pT18mob2 | Tet^r^, pK18*mob*2 derivate | [7] |
| pK19mobGII | Km^r^, pMB-1 replicon, *gusA*, *lacZα* | [1] |
| pCM184 | Km^r^, Ap^r^, Tet^r^ | [4] |
| pCM157 | Tet^r^, Cre expression vector | [4] |
| pBBR-MCS2 | Km^r^, *lacZa* | [8] |
| pBBR-MCS5 | Gm^r^, *lacZa* | [8] |
| pAP150 | pBBR-MCS2, P_mamDC45_, *gfp2*, terminator sequence from pUC18R6K | [9] |
| pAS200 | Gm^r^, ColE1 ori, *sacB* of *Bacillus subtilis* | [4] |
| pAL01 | pK19mobGII digested with *SalI* and *EcoRI*, insertion of *lox71* and homologous sequence AL01 | this study |
| pAL01_MCS1 | pAL01, digested with *EcoRI* and *NotI*, insertion of MCS from pBBR-MCS5 | this study |
| pT18mob2_AL02/2 | pT18*mob*2 digested with *SalI* and *HindI*, insertion of the *lox66* and homologous sequence AL02/2 | this study |
| pAL02/2_Tet | pT18*mob*2_AL02/2 digested with *SalI* and *EcoRI*, insertion of gentamicin gene from pBBR-MCS5 | this study |
| pAL02/2 | pAL02/2_Tet digested with *PstI*, blunted and self-ligated | this study |
| pAL02/2_MCS2 | pAL02/2 digested with *HindIII* and *BamHI*, insertion of MCS from pBBR-MCS5 | this study |
| pAL02/2_term | pAL02/2_MCS2 digested with *KspI*, insertion of terminator sequence | this study |
| pAL03 | pAL01_MCS1 digested with *ClaI* and *NotI*, insertion of homologous sequence AL03 | this study |
| pAL05 | pAL01 digested with *EcoRI* and *NotI,* insertion of homologous sequence AL05 | this study |
| pAL06 | pAL02/2_MCS2 digested with *PvuI* and *XhoI*, insertion of homologous sequnce AL06 | this study |
| pAL07 | pAL01 digested with *EcoRI* and *NotI*, insertion of homologous sequence AL07 | this study |
| pAL08 | pAL02/2 digested with *BamHI* and *NotI*, insertion of homologous sequence AL08 | this study |
| pAL11_term | pAL02/2_term digested with *BamHI* and *NotI*, insertion of homologous sequence AL11 | this study |
| pCM184_mms6_5' WT | pCM184 digested with *MfeI* and *NdeI*, insertion downstream fragment of *mgr4070* | this study |
| pCM184_mms6_5'3' WT | pCM184_mms6_5' WT digested with *ApaI* and *SacI*, insertion upstream fragment of *mgr4074* | this study |
| pCM184_mms6_5'3' GFDC | pCM184_mms6_5' WT digested with *MluI* and *SacI*, insertion downstream fragment of *mamC* | this study |
| pCM184_mamXY_5' | pCM184 digested with *MfeI* and *NcoI*, insertion downstream fragment of *ftsZm* | this study |
| pCM184_mamXY_5'3' | pCM184_mamXY_5' digested with *ApaI* and *SacI*, insertion upstream fragment of *mamX* | this study |
| pCM184_mamXY_5'SU | pCM184 digested with *EcoRI* and *SmaI*, insertion downstream fragment of *ftsZm* | this study |
| pCM184_mamXY_5'3'SU | pCM184 digested with *ApaI* and *ClaI*, insertion upstream fragment of *mamX* | this study |
| pCM184_mamW_5' | pCM184 digested with *MnuI* and *NdeI*, insertion upstream fragment of *mamW* | this study |
| pCM184_mamW_5'3' | pCM184_mamW_5' gigested with *ApaI* and *SacI*, insertion downstream fragment of *mamW* | this study |
| pSUMAI13_3' | pAS200 digested with *SalI* and *HindIII*, insertion upstream fragment of *mgr4174* | this study |
| pSUMAI13_5' | pKmobGII digested with *BamHI* and *XbaI*, insertion upstream fragment of *mgr4151* | this study |
| pK19mobGII_mamJKL_3' | pK19mobGII digested with *XbaI* and *SpeI*, insertion of upstream fragment of *mamJ* | this study |
| pK19mobGII_mamJKL_3'5' | pK19mobGII_mamJKL_3' digested with *SmaI*, insertion of downstream fragment of *mamL* | this study |
| pCDS52_mms6_mmsF | pBBR-MCS2 digested with *NsiI* and *EcoRI*, insertion *mmsF*, *mms6* and *mgr4074* | this study |
| pmamXY | pBBR-MCS2 digested with *NsiI* and *EcoRI*, insertion of *mamXY* operon, native promotor | this study |

1. Katzen F, Becker A, Ielmini MV, Oddo CG, Ielpi L (1999) New mobilizable vectors suitable for gene replacement in gram-negative bacteria and their use in mapping of the 3' end of the Xanthomonas campestris pv. campestris gum operon. Appl Environ Microbiol 65: 278-282.

2. Scheffel A, Gardes A, Grünberg K, Wanner G, Schüler D (2008) The major magnetosome proteins MamGFDC are not essential for magnetite biomineralization in Magnetospirillum gryphiswaldense but regulate the size of magnetosome crystals. J Bacteriol 190: 377-386.

3. Schultheiss D, Schüler D (2003) Development of a genetic system for Magnetospirillum gryphiswaldense. Arch Microbiol 179: 89-94.

4. Marx CJ, Lidstrom ME (2002) Broad-host-range cre-lox system for antibiotic marker recycling in gram-negative bacteria. Biotechniques 33: 1062-1067.

5. Ullrich S, Schüler D (2010) Cre-lox-based method for generation of large deletions within the genomic magnetosome island of Magnetospirillum gryphiswaldense. Appl Environ Microbiol 76: 2439-2444.

6. Schübbe S, Kube M, Scheffel A, Wawer C, Heyen U, et al. (2003) Characterization of a spontaneous nonmagnetic mutant of Magnetospirillum gryphiswaldense reveals a large deletion comprising a putative magnetosome island. J Bacteriol 185: 5779-5790.

7. Tauch A, Kirchner O, Löffler B, Götker S, Pühler A, et al. (2002) Efficient electrotransformation of corynebacterium diphtheriae with a mini-replicon derived from the Corynebacterium glutamicum plasmid pGA1. Curr Microbiol 45: 362-367.

8. Kovach ME, Elzer PH, Hill DS, Robertson GT, Farris MA, et al. (1995) Four new derivatives of the broad-host-range cloning vector pBBR1MCS, carrying different antibiotic-resistance cassettes. Gene 166: 175-176.

9. Pollithy A, Romer T, Lang C, Muller FD, Helma J, et al. (2011) Magnetosome expression of functional camelid antibody fragments (nanobodies) in Magnetospirillum gryphiswaldense. Appl Environ Microbiol.
