# Supplementary material for: Functional Analysis of the Magnetosome Island in Magnetospirillum gryphiswaldense: The mamAB Operon Is Sufficient for Magnetite Biomineralization
Source: PLoS One. 2011 Oct 17;6(10):e25561. doi: 10.1371/journal.pone.0025561 (PMC3197154; doi:10.1371/journal.pone.0025561)
Supplement: Table S3 — Annotation and characteristics of MAI genes of M. gryphiswaldense. (DOC) [file pone.0025561.s005.doc]

**Table S3. DNA oligonucleotides used in this work.**

| Name | Sequence |
| --- | --- |
| AL019 | 5'-ATTGTCGACATAACTTCGTATAGCATACATTATACGAACGGT AGGATCCGGCATCCTGATCGGTAGGCGAT |
| AL020 | 5'-AAGCTTAGAAGGGTTACGACGCCGGT |
| AL033 | 5’-GAATTCGGCTGTTCGGCACCTCTGTT |
| AL034 | 5’-AATGTCGACTCTAGACTCGAGATAACTTCGTATAATGTATG CTATACGAACGGTAGCGGCCGCTGATCTCGGGATCACTCGGT |
| AL042 | 5'-GAATTCGCCACCTTGACAGAAATTGATATC |
| AL043 | 5'-GCGGCCGCTCTTCCAACGAAATTGTGCG |
| AL048 | 5'-GAATTCTTACCGCTCTTCGGCATCCACGCC |
| AL049 | 5'-GCGGCCGCGGCAGCCTCATTTAAACATTCAGG |
| AL081 | 5'-GAATTCACACCGTGGAAACGGATGAAGGCAC |
| AL082 | 5'-GTCGACGCGCCTGAAGCCCGTTCTGG |
| AL092 | 5'-GGATCCATGCCGGCGGACAGCAGATGCT |
| AL093 | 5'-GCGGCCGCGCACGGAGACTCTCATAGTG |
| AL094 | 5’-GGATCCACGCCTTCATCCTTGAACCA |
| AL095 | 5’-GCGGCCGCTGGACATCAACGAAAAGGCA |
| AL107 | 5'-ATCGATGTTCGATATCCGTCTGCGC |
| AL108 | 5' - GCGGCCGCCTCTTCGGGGCAACGTGAA |
| AL113 | 5'-GGATCCATAAGAATGCGGCCGCCCGCTCGAGCCCGGGCT GCAGGAATTCGA |
| AL114 | 5'-AAGCTTTGGGTTCGTGCCTTCATCCG |
| AL115 | 5'-GAATTCGGATCCGATATCAAGCTTATCGATACCGTCGACC |
| AL116 | 5'-GCGGCCGCTGGGTTCGTGCCTTCATCCGTT |
| AL121 | 5'-CTCGAGTATCGCCACCTTATGGGGAG |
| AL122 | 5'-CGATCGATGCTGTCGGCCATCATCAT |
| AL125 | 5'-GAATTCCTGATCTCCGGCAAGTGTAT |
| AL132 | 5'-ACGCGTTGAAATATTGGGCTGGTTCACG |
| AL133 | 5'-GAGCTCTGCTGCTGCCAATATCGTCG |
| AL136 | 5'-ATGCATTCACCCGAGGCCGAACCTCA |
| AL152 | 5'-TATACCGCGGGGCGGATTTGTCCTACTCAGG |
| AL153 | 5'-GACTCCGCGGGACTCCTGTTGATAGATCCAGTAATGAC |
| AL178 | 5'-GGATCCTTCATGTACTGCGGAACAGTCG |
| AL179 | 5'-CATATGTTGGGCTTGTGGTTTTGGCGG |
| AL188 | 5'-GGGCCCAAGGGCTGCTCCCGTGGTGG |
| AL189 | 5'-GAGCTCCCCACGCATGTACACAGCCATA |
| AL190 | 5'-CAATTGCTCGCTAAAAATGTGGGTTTCCG |
| AL191 | 5'-CCATGGGCCGCTCCGGAAGAATCAAGC |
| AL352 | 5'-CAATTGTGGCCCCGGTCAAGTCAACT |
| AL353 | 5'-CATATGTACATGAGGGCATCGCGTTG ' |
| AL354 | 5'-GGGCCCAATTGTCGACAAATCCCAAAGA |
| AL355 | 5'-GAGCTCCCAAAGCAAAGGACTCCG |
| SU88 | 5'-GAATTCTAAAAATGTGGGTTTCCG |
| SU89 | 5'-CCCGGGTGAGCCGCTCCGGAAGAAT |
| SU304 | 5'-CAATTGATTGCCAGATGATCTTGATCATGTC |
| SU305 | 5'-CATATGCATGGCCGCTTTCCAAACAGGTGA |
| SU306 | 5'-GGGCCCTGAGGGAGTGGGGACTGCGAAGTA |
| SU307 | 5'-GAGCTCAGCCGAACCGACCAAATACTGG |
| SU422 | 5'-ATCGATCACAAGGGATAGATATGGC |
| SU423 | 5'-GGGCCCCTAACCTTGATCCCCG |
| SU488 | 5'-GTCGACATGCCGGCGGACAGCAGATGCT |
| SU489 | 5'-AAGCTTGCACGGAGACTCTCATAGTG |
| SU510 | 5'-GGATCCTTACCGCTCTTCGGCATCCACGCC |
| SU511 | 5'-TCTAGACAAGGCAGCCTCATTTAAACATTCAGG |
| EK1 JKL u_f | 5'-TCTAGAGCAGCCGGTCCGATCGCCTTTGG |
| EK_JKL u_r | 5'-CCCGGGATTGCTAACTAGTTATCCCGCTCCACCCTCAAAGAA |
| EK_JKL d_f | 5'-ACTAGTCGCATTCCATGCTCCGTCGGAGC |
| EK2_JKL d_r | 5'-CCCGGGGGATCCCCGACGAAAATGGTTACGCCCG |
