# Supplementary material for: Functional Analysis of the Magnetosome Island in Magnetospirillum gryphiswaldense: The mamAB Operon Is Sufficient for Magnetite Biomineralization
Source: PLoS One. 2011 Oct 17;6(10):e25561. doi: 10.1371/journal.pone.0025561 (PMC3197154; doi:10.1371/journal.pone.0025561)
Supplement: Materials and Methods S1 — Construction of integrative plasmids and deletion mutagenesis/Conjugation experiments. (DOC) [file pone.0025561.s006.doc]

**Materials and Methods**

Construction of integrative plasmids and deletion mutagenesis

Downstream and upstream sequences of the deletion targets (AL01-AL03; AL05-AL08; AL11; Fig. S2) were amplified by PCR using *M. gryphiswaldense* chromosomal DNA and oligonucleotides listed in Table S3. PCR products were subcloned into pJet1.2 vector, sequenced and finally ligated into the mobilizable suicide plasmids. The basic suicide vector, pAL01 was constructed by amplifying homologous region AL01 by PCR using the primer pair AL33/AL34, containing the *lox71* sequence. AL01 was digested with *EcoRI*-*SalI* and inserted into the corresponding site of pK19mobGII [1], resulting in pAL01. After digestion with *EcoRI* and *NotI* the plasmid was used for constructing vectors pAL05 and pAL07. The homologous regions were amplified with primers AL42/AL43 and AL48/AL49, respectively. Moreover, the multiple cloning site (MCS) from pBBR-MCS5 plasmid was amplified by PCR with AL115/AL116 primers. The fragment was digested with *EcoRI*-*NotI* and ligated into the same position of pAL01, creating pAL01_MCS1. Consequently, the homologous region AL03, amplified with primers AL107/AL108, was integrated after digestion with *ClaI* and *NotI*, resulting in pAL03. The basic vector pAL02/2 was constructed amplifying the homologous sequence AL02/2 by PCR using the primer pair AL19/AL20, containing the *lox6*6 site. The 2148-bp fragment was cut with *SalI-HindIII* and cloned into pT18mob2. The resulting plasmid was designated pT18mob2_AL02/2. Gene for gentamicin resistance (Gm) was amplified by PCR from pBBR-MCS5 plasmid with primers AL81/AL82, and was inserted after digestion with *EcoRI-SalI*, resulting in pAL02/2_Tet. Tetracycline resistance gene was destructed by digestion with *PstI* and the blunted and self-ligated vector was named pAL02/2. The MCS from pBBR-MSC5 was amplified with primers AL113/AL114 and the fragment was cut with *HindIII*-*BamHI*. Thus, the following plasmids were generated by using the following primer pairs and restriction endonucleases: pAL06 (AL121/AL122; *XhoI-PvuI*) as well as pAL08 (AL92/93; *BamHI- NotI*). Due to plasmid instability a terminator sequence was inserted into pAL02/2_MCS2 after amplification with primers AL152/AL153 from plasmid pAP150 and digestion with *KspI*, resulting in pAL02/2_term. The plasmid was used to construct pAL11_term, whereby homologous sequences were amplified with primer pair AL94/95 and digested with *BamHI-NotI*. Excisions of the *mms6* operon and *mamXY* operon were conducted by double cross-over mutagenesis as described previously [2,3]. Consequently, pCM184 [4] derivate were generated, whereby following oligonucleotides and restriction endonucleases were used to amplify and insert corresponding downstream and upstream fragments: pCM184_mms6_5'3' WT ([AL352/AL353; *MfeI*-*NdeI*], [AL354/AL355; *ApaI*-*SacI*]); pCM184_mms6_5'3' GFDC ([AL352/AL353; *MfeI*-*NdeI*], [AL132/AL133; *MluI-SacI*]); pCM184_mamXY_5'3' ([AL190/AL191; *MfeI*-*NcoI*], [AL188/AL189; *ApaI*-*SacI*]) and pCM184_mamXY_5’3’SU ([SU88/Su89; *EcoRI-SmaI*], [SU422/423; *ApaI-ClaI]*). While pCM184_mamXY_5'3' was used in the ΔA12 background, pCM184_mamXY_5’3’SU was employed in wildtype and Δ*GFDC* [2]. For Single gene excision of the *mamW* gene, upstream fragment was PCR amplified using primer pairs SU304/SU305 and SU306/SU307 for downstream region. Constructs were digested with *MunI*-*NdeI* or *ApaI*-*SacI* and ligated into pCM184, resulting in pCM184_mamW_3’5’. After conjugation of the final integrative plasmids into *M. gryphiswaldense* strains, single or double insertion mutants were selected with corresponding antibiotics and verified by direct cell PCR. The excision of large genomic segments was induced after conjugation with the Cre expression plasmid pCM157 [4]. Original lac promotor was exchanged by a native *M. gryphiswaldense* promotor (generated by Y. Le, unpublished data). To obtain marker-less mutants after double crossover occurred, the vector was also used for deletion of the inserted *Km* gene from pCM184. Specific gene replacements were verified by PCR and sequencing. ΔA19 was designed as previously reported [5] using MSR-1B as parent strain and the plasmids pSUMAI13_5’ (pK19mobGII derivate) and pSUMAI13_3’ (pAS200 derivate) generated with following primer pairs and matching restriction endonucleases: [SU510/SU511; *BamHI*/*XbaI*] and [SU488/SU489; *SalI*/*HindIII*]. Plasmid pK19mobGII_mamJKL_3’5’ for deletion of genes *mamJ,K,L* was generated by inserting 1 kb fragments upstream and downstream of *mamJ* and *mamK* (amplified with primer pairs EK1_JKL u_f/ EK1_JKL u_r and EK_JKL d_f/ EK_JKL d_r) into pK19mobGII after digestion with *XmaI*-*SpeI* and *SmaI*, respectively.

Conjugation experiments

Plasmid transfer via conjugation was performed with *E. coli BW29427* as donor strain and *M. gryphiswaldense* R3/S1 or its descendants as acceptor strains. Conjugation procedure was performed as described previously [3,5] with following modifications for genomic plasmid insertion: After the first plasmid transfer and 2h cultivation in liquid media, cells of single insertion mutants were grown in 100 ml FSM under selective conditions. *E. coli BW29427* containing the second insertion plasmid, was added after 32h of incubation. The concentrated suspension was spotted onto FSM agar dishes containing DAP, incubated for 8h and flushed from agar surface. After incubation of 2h in liquid FSM, cells were grown on selective agar plates containing X-Gluc. Blue colonies were transferred in 100 µl FSM as well as scaled up to 10 ml after positive testing for plasmid integration via PCR. Double integration mutants were subjected to excision by conjugation with pCM157 [5].

For *trans*-complementation, plasmid pCDS52_mms6_mmsF containing *mmsF*, *mms6*, *mgr4074* and the native *mms6* promoter (P_mms6_) was constructed. The 1,448 bp fragment was digested with *NsiI*-*EcoRI*, and inserted into the same sites of pBBR-MCS2. Plasmid pmamXYop was constructed by PCR amplification of the *mamXY* operon with primer pairs AL200/AL201, which was inserted into pBBR-MCS2 after digestion with *NdeI* and *XbaI.* Other mutants could not be complemented because of their large sizes between 6 and 68 kb.
